# New Insights into the Fanconi Anemia Pathogenesis: A Crosstalk Between Inflammation and Oxidative Stress

**DOI:** 10.3390/ijms252111619

**Published:** 2024-10-29

**Authors:** Anna Repczynska, Barbara Ciastek, Olga Haus

**Affiliations:** 1 Department of Clinical Genetics, Faculty of Medicine, Collegium Medicum in Bydgoszcz, Nicolaus Copernicus University in Torun, Curie Sklodowskiej 9, 85-094 Bydgoszcz, Poland; haus@cm.umk.pl; 2 Institute of Health Sciences, University of Opole, Katowicka 68, 45-060 Opole, Poland

**Keywords:** Fanconi anemia, inflammatory process, autophagy, oxidative stress, bone marrow failure, cancers

## Abstract

Fanconi anemia (FA) represents a rare hereditary disease; it develops due to germline pathogenic variants in any of the 22 currently discovered *FANC* genes, which interact with the Fanconi anemia/breast cancer-associated (FANC/BRCA) pathway to maintain genome integrity. FA is characterized by a triad of clinical traits, including congenital anomalies, bone marrow failure (BMF) and multiple cancer susceptibility. Due to the complex genetic background and a broad spectrum of FA clinical symptoms, the diagnostic process is complex and requires the use of classical cytogenetic, molecular cytogenetics and strictly molecular methods. Recent findings indicate the interplay of inflammation, oxidative stress, disrupted mitochondrial metabolism, and impaired intracellular signaling in the FA pathogenesis. Additionally, a shift in the balance towards overproduction of proinflammatory cytokines and prooxidant components in FA is associated with advanced myelosuppression and ultimately BMF. Although the mechanism of BMF is very complex and needs further clarification, it appears that mutual interaction between proinflammatory cytokines and redox imbalance causes pancytopenia. In this review, we summarize the available literature regarding the clinical phenotype, genetic background, and diagnostic procedures of FA. We also highlight the current understanding of disrupted autophagy process, proinflammatory state, impaired signaling pathways and oxidative genotoxic stress in FA pathogenesis.

## 1. Introduction

### 1.1. Definition of FA

**Figure 1:**
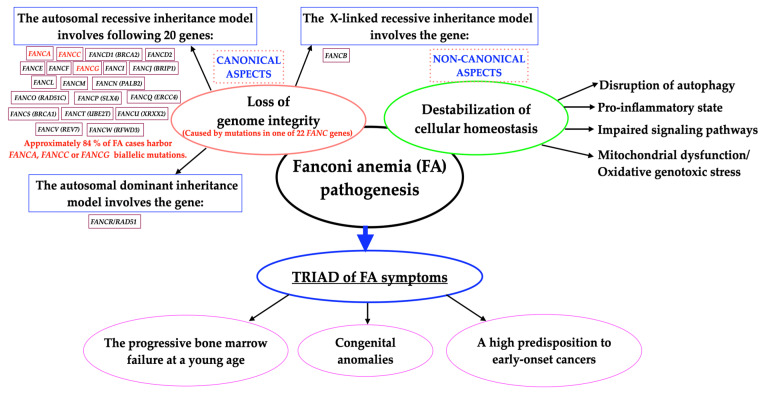
Schematic concept of Fanconi anemia pathogenesis.

Fanconi anemia (FA) is a rare (1:160,000), principally autosomal recessive hereditary disease (except for *FANCB* and *FANCR/RAD51)* (Figure 1); it develops due to biallelic germline downregulation of any one of the 22 currently identified *FANC* complementation genes (*FANCA–FANCW*) [1,2,3,4,5]. Moreover, the following eight proteins, FAAP10, FAAP16, FAAP20, FAAP24, FAAP100, UHRF1/2, USP1/UAF1 and FANI, are engaged in the FA pathway [6]. The FANC/BRCA pathway exhibits the canonical FA signaling transduction pathway [7]. Furthermore, FA proteins exert noncanonical effects by regulating mitochondrial metabolism, inflammation and redox balance in a DNA repair-independent manner [8].

The cellular hallmarks of FA include impaired repair of DNA interstrand cross-linking (ICLs), disruption of DNA replication fork, impaired autophagy and mitophagy (the autophagy of damaged mitochondria), overproduction of proinflammatory cytokines and dysregulation of the redox system [2,9,10,11,12,13]. FA is also characterized by cellular hypersensitivity to exogenous ICL agents, including diepoxybutane (DEB), mitomycin C (MMC), cisplatin, tobacco smoke, etc. [5,7]. Combining all of the above leads to the manifestation of a triad of FA symptoms, including the early onset of bone marrow failure (BMF), a high predisposition to a wide spectrum of malignancies and congenital anomalies [11,14,15]. In addition, FA is the most common form of inherited bone marrow failure syndromes (IBMFS) and chromosome instability syndrome associated with developmental anomalies [16]. Figure 1 summarizes the general concept of FA pathogenesis, including all FA genes, the inheritance pattern, and the two pillars influencing the development of the disease, i.e., loss of genome integrity and destabilization of cellular homeostasis.

### 1.2. Clinical Presentation

FA patients demonstrate multiple congenital anomalies, including short stature, microcephaly, hydrocephalus, absent radius, thumb hypoplasia or aplasia, abnormal skin pigmentation, as well as other organ abnormalities most frequently found in the urinary and cardiovascular systems [4,16]. Some FA patients are adults with a normal general appearance and a normal complete blood count [17]. However, 30% of FA patients develop BMF in childhood and approximately 7% of patients progress to myeloid malignancies by the age of 18 [5]. Patients with FA show a 700- and 6000-fold increased risk of acute myeloid leukemia (AML) and myelodysplastic syndrome (MDS), respectively, compared to healthy counterparts [6,18]. These patients are diagnosed at the end of the first decade of life; however, some patients are diagnosed in adulthood [16,19]. Approximately one-third of patients have no overt hematological abnormalities. Furthermore, in adults with FA, the development of squamous-cell carcinoma (SCC) is the most life-threating complication. The overall risk of SCC in FA patients is 500-fold higher than in the healthy population [20]; the mean age at diagnosis of SCC is 15–16.5 years in patients with FA compared to 45–54 years in those without FA [21]. Due to a defect of DNA repair systems, FA patients cannot tolerate standard SCC chemoradiotherapy and treatment side effects are hard to predict [22].

Research endeavors have highlighted substantial heterogeneity in FA development and phenotype, even among patients harboring the same pathogenic variant (PV). Probably other factors may contribute to both hematologic and non-hematologic manifestations of FA, including inflammatory cytokine production, oxidative stress, impaired mitochondrial metabolism or epigenetic factors [23,24]. Additionally, FA is also called a premature-aging state, with high rates of stem cell depletion at birth, high cancer prevalence and a general decline in physical fitness which appear at a young age [25]. Thus, the heterogeneity of the clinical picture of FA may result in a delay in establishing the definitive diagnosis, which strongly affects the future patients’ outcomes [17]. A positive result of the chromosome breakage test confirms the clinical diagnosis of FA and is also an indication to extend the diagnostics with the molecular cytogenetics and molecular methods in order to precisely determine the genetic background of the disease, and detect the type and origin of PV and neoplastic to adequately carry out patients in genetic council clinics [26,27].

The procedure that rescues against the consequences of BMF is hematopoietic stem cell transplantation (HSCT) [28]; however, this treatment does not protect against the pro-neoplastic proneness in FA patients, since these patients still demonstrate an extremely high risk of head and neck (HNC), esophagus, gastrointestinal tract, vulvar or anus cancers [7,16,21,29,30]. Furthermore, FA-related cancers display an aggressive phenotype; therefore, FA patients require early medical evaluation and implementation of personalized and more efficient treatment [7,16,21,29]. Herein, we review and discuss the general definition, clinicopathologic characteristics and diagnostic technologies in FA. Further, we summarize and update the current understanding of disrupted autophagy process, proinflammatory state, impaired signaling pathways and oxidative genotoxic stress in the pathogenesis of FA.

## 2. Genetic Background of FA

The human *FANCC* gene was first identified and its cDNA was cloned in 1992 by Strathdee et al. [30]. In the following years, the presence of subsequent 21 FA disease-causing genes (*FANCA–FANCW*) was confirmed [2,3,4,5,6]. Delving deeper into the pathogenesis of FA, we need to account the proteins encoded by the *FANC* genes, of which three groups of proteins involved in DNA metabolism can be distinguished. Therefore, the canonical FA signaling pathway is commonly divided into three components [7]. Group I proteins are linked to Fanconi anemia-associated protein (FAAP) proteins and create the FANC nuclear core complex, which works as a ubiquitin ligase. This nucleus complex mediates monoubiquitylation of the FANCD2 and FANCI, two members of heterodimer ID complex (group II) during the S phase of the cell cycle. The third FANC group proteins contribute to DNA repair, and cell-cycle regulation for the repair of DNA ICLs (emerged in the presence of reactive oxygen species (ROS) or aldehyde substrates), in order to preserve genome integrity. ICLs are covalent adducts between DNA strands, which diminish transcription and replication and may promote the formation of double-strand DNA breaks, the most detrimental types of DNA lesions. To extend cell viability, permanent eradication of ICL is necessary [3,6,8,12,13,23,24,25,26,27,28,29,30,31,32,33,34]. Persistent DNA damage (genotoxic stress) triggers signaling cascades that drive cells toward apoptosis or senescence to avoid replication of the damaged genome. The downside of this state is that these cancer evasion mechanisms promote cells senescence [35]. Additionally, selected FA proteins regulate cellular homeostasis, including protection against inflammation-induced apoptosis, maintenance of mitochondrial integrity and inhibition of intracellular ROS generation [8,9,10,11,12,13]. In Table 1, we demonstrate the current status of the *FANC* gene library, the *FANC* gene loci with FA protein functions as well as the frequency of pathogenic variants and the inheritance pattern [6,7,8,26,33,34,36]. Furthermore, Engel et al. demonstrated how pathological activation of the central DNA repair system paradoxically triggers the evolution of the cancer genome through an event called chromothripsis. This recent finding highlights that FA pathway-induced chromothripsis generates complex genomic and extrachromosomal DNA rearrangements that confer acquired resistance to anticancer therapies [37].

## 3. Methods of FA Detection and Evaluation

**Figure 2:**
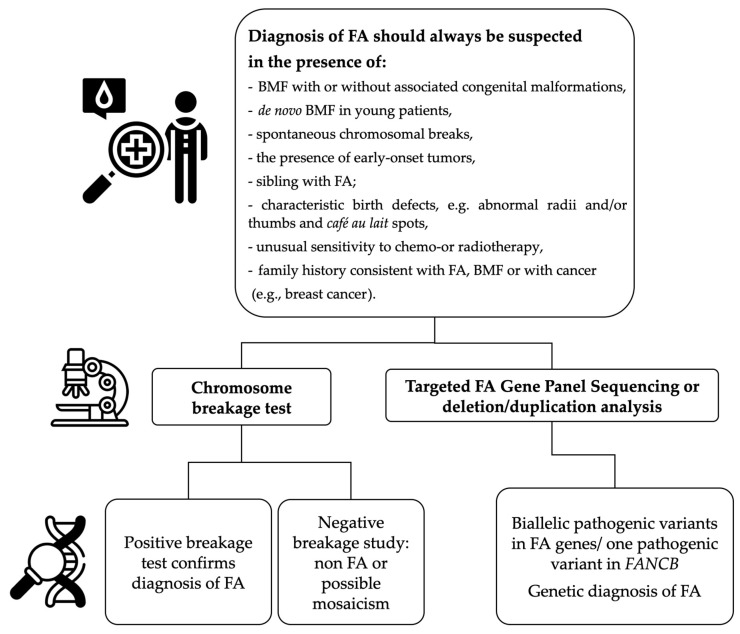
A simplified diagnostic algorithm for Fanconi anemia, including indications for FA diagnosis and general genetic tests.

Diagnosis of FA should be established in highly specialized centers and the highest quality laboratories. The diagnostic algorithm (Figure 2) for patients suspected of FA, in addition to the sequencing method, also includes the analysis of DNA copy number variants (CNVs) using molecular cytogenetics techniques, such as multiplex ligation-dependent probe amplification (MLPA) and microarray comparative genomic hybridization (aCGH) or, as a new option—copy number variant (CNV) testing with NGS. CNV analysis is necessary to detect large gene rearrangements: deletions, duplications and insertions. Large deletions are detected in approximately 35% of patients with Fanconi anemia, which constitute 18% of all PVs detected in the result of molecular testing in FA patients [36].

Variants in FA complementation group A (*FANCA*) are the most frequent cause of FA and are responsible for about 60% of cases. The role of the novel protein product of this gene is recruiting a DNA repair system to areas of DNA damage. *FANCA* variants include single nucleotide variants, small insertions and deletions which may be detected by next-generation sequencing techniques (NGSs). Missense PV stand for about 15% of all pathogenic changes. However, 20–40% of *FANCA* PVs are large deletions, of which their size spans a wide range from ~1 kb to 545 kb, detected by aCGH or MLPA [27]. PV of the *FANCA* gene in patients with Fanconi anemia indicates the possibility of premature ovarian insufficiency (POF) beyond the classical triad of symptoms [38]. The next most frequent PVs that are responsible for about 20% of cases are *FANCC* and *FANCG*. Causative PVs in other FA genes are rare and account for 0.1–3% of cases per gene [2]. Table 2 presents a brief description of the applications, advantages and limitations of currently used FA diagnostic technologies [26,34,36,39].

Although the classic chromosome breakage test has some limitations, it still remains the gold standard for FA diagnosis. It is based on the hypersensitivity of FA-derived cells to ICLs agents, including MMC, cisplatin or DEB. Lymphocytes from peripheral blood samples of FA patients are stimulated by T-cell mitogen phytohemagglutinin (PHA), cultured with or without MMC/DEB (control) and harvested. DNA damage effects are analyzed in metaphases spreads [17,26,27,39]. Typically, chromosome aberrations are gaps, chromatid/chromosome breaks, acentric fragments, dicentric chromosomes, ring chromosomes and characteristic radial figures [26,27]. Radial figures are damage-induced chromosomal aberrations that rarely occur spontaneously. It has been reported that radials can be induced by DNA double-strand breaks (DSBs) that are generated by exposure to ICL-inducing agents, including ionizing radiation (IR) and replication inhibitors. However, despite the fact that these DSB inducers can (albeit rarely) cause the formation of radial lesions, the requirement of the probable presence of at least two DSBs in two or more non-homologous chromosomes still leaves the unanswered question, which DSB repair pathway generates radial lesions? Rogers et al. showed that radials observed in *FANCD2*^−/−^ cells are dependent on POLθ and DNA ligase III and occur independently of classical nonhomologous end joining. Furthermore, treatment of *FANCD2*^−/−^ cells with POLθ inhibitors abolishes radials and leads to the accumulation of breaks co-localizing with common fragile sites. Moreover, these observations implicate A-EJ in radial formation and provide mechanistic insights into the treatment of FA pathway-deficient cancers with POLθ inhibitors [40].

**Figure 3:**
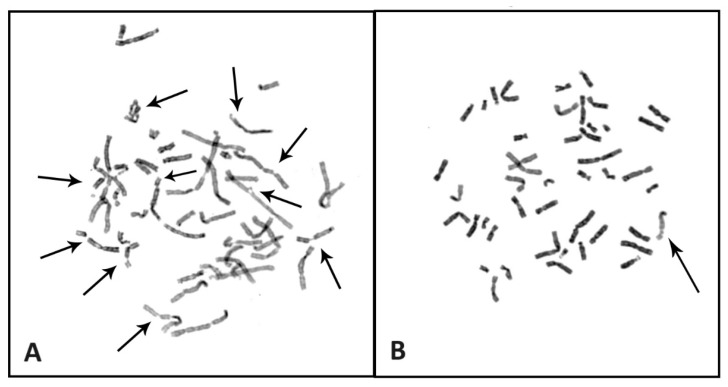
Chromosomal aberrations after addition of cross-linking agent (MMC) to the lymphocyte culture of patient with FA (**A**) and healthy control (**B**). Arrows indicate chromatid breaks.

Figure 3 shows examples of metaphase spreads from a patient with FA (picture A) and a healthy subject (picture B). Similar tests can be performed in amniocytes and fibroblasts. Chromosome breakage test is not 100% specific for FA and can produce false-positive results in patients with other chromosome instability syndrome, e.g., Nijmegen syndrome or Warsaw breakage syndrome [26,27,34,39]. Increased chromosome breakage and the above-mentioned chromosome aberrations may be absent or present in only a small percentage of cells in approximately 10–20% of patients with Fanconi anemia, presenting with somatic hematopoietic mosaicism, which is characterized by a parallel presence of cells insensitive and sensitive to DNA ICL agents (MMC, DEB). From this point of view, it is crucial that a laboratory determines the reference values for positive, intermediate and negative results of the chromosome breakage test performed in patients representing a population with suspected Fanconi anemia and a control population [34,39,41].

Summing up, the assessment of the genetic background of Fanconi anemia is a complex research and diagnostic process that includes cytogenetic, cytogenetic–molecular and molecular methods. The obtained results make it possible to determine the level of chromosome breaks and the presence of characteristic aberrations in cells suffering from Fanconi anemia.

Finally, the detection of new large deletions, duplications and PVs, which play a role in the understanding of pathogenesis of this rare disease, which in the context of the rarity and heterogeneity of Fanconi anemia has cognitive value. Intriguingly, the FANC/BRCA pathway proteins also participate in noncanonical regulation of cellular homeostasis, including immune response, autophagy, redox regulation and mitochondrial metabolism; thus, expanding knowledge in this field is warranted.

## 4. Brief Description of FA Treatment

Despite the well-established knowledge of the complex pathogenesis and diagnostic procedures in FA, there is still an urgent need to find effective treatment for BM failure in FA patients and their efficient implementation in clinical practice. The treatment strategies for FA encompass generally hematopoietic stem cell transplantation, androgene therapy, gene therapy, targeted medications and supportive care based on novel targeted chemoprevention therapy. These treatment armamentarium have significantly improved the efficacy, accuracy and long-term follow-up of FA patients. However, clinical trials of FA gene therapy are still insufficient due to limited study populations and short follow-ups. However, the role of danazol in telomere disorders, with regard to its optimal dose, safety, effectiveness and long-term effects is currently being analyzed (2024) in clinical trials in the United States and France (clinicaltrials.gov identifiers: NCT03312400, NCT03312400) [42]. Independently, there arises the question whether natural redox regulators, including quercetin, green tea (catechins) and resveratrol could be included in the therapy of FA patients as an adjuvant treatment. Clinical trials of long-term oral quercetin to enhance HSCs function in FA patients without previous HSCT (ClinicalTrials.gov Identifier: NCT01720147, accessed on 11 March 2022) and to prevent HNC (ClinicalTrials.gov Identifier: NCT03476330, accessed on 11 March 2022) are ongoing in Cincinnati Children’s Hospital Medical Center [1]. Numerous clinical trials are being conducted to improve the quality of life of patients suffering from FA, as the current treatment strategies are unsatisfactory. Table 3 presents the general aspects of the management of patients with FA, their indications, advantages and limitations [1,26,42,43,44,45,46,47,48,49,50,51,52].

## 5. Deep Insight into FA Pathogenesis

### 5.1. Destabilization of Cellular Homeostasis via Disruption of Autophagy Process

The loss of cellular homeostasis is closely related to autophagy process, since this process is responsible for cell protection, maintaining an anti-inflammatory environment, excretion of unfavorable products, which arose during physiological cell metabolism, and eradication of intracellular microorganisms [9,53]. The accumulation of DNA damage triggers the process of autophagy to defend the cell from damage and preserve its survival. Interestingly, autophagy components are able to stimulate the secretion of proinflammatory interleukin 1β (IL-1β) from the cytoplasmic matrix into the extracellular space, where IL-1β exerts its biological function [54]. Moreover, the autophagy process regulates the nuclear factor kappa B (NF-κB) pathway and thus suppresses inflammation in tissues [53]. However, a disturbed autophagy process is associated with excessive inflammatory response and tissue damage. Furthermore, autophagy collaborates with signaling pathways, including Wnt and Notch, which gives it space to influence the regulation of cell cycle [9,53,55]. The regulation of cell proliferation, differentiation, maturation, motility and apoptosis depends on the Notch signaling pathway. Interestingly, an imbalance in the Fanconi pathway dysregulates cellular integrity and reduces proliferation capacity [9]. Zipporah et al. demonstrated the role of autophagy in Notch signaling, and its interrelated outcome in transmitting the cell proliferation impulse in FA. Moreover, both pathways are persistently disrupted in FA; Notch signaling and autophagy genes are negatively correlated. Thus, stimulation of autophagy regulates the Notch signaling pathway and enhances the cellular lifespan in FA [9]. Shyamsunder et al. showed that FA-derived cells accumulate numerous autophagic (possibly mitophagic) events and an increased number of pathological mitochondria due to a disturbed mitochondria elimination process leading to a shift towards the activation of oxidative stress [55]. Last but not least, Zipporah et al. claimed that in FA, impaired autophagy preserves the processed intracellular Notch receptor domain inside the cells, which promotes *HES1* family genes transcription, whereas upregulated *HES1* gene expression is commonly linked to the pro-neoplastic state [9].

### 5.2. Destabilization of Cellular Homeostasis via Over-Activation of Inflammatory Process and Impaired Signaling Pathways

The cellular response to any loss of tissue integrity can trigger an inflammatory reaction, in order to preserve homeostasis. The inflammatory process is tightly regulated. However, its dysfunction is believed to be one of the causes of a dynamic cascade starting with impaired tissue repair, ending with collateral damage and ultimately pathological conditions development [7,8,56]. Inactivation or downregulation of the *FANC* genes leads to a loss of genome integrity, which results in the higher proneness to solid or hematological malignancies due to the accumulation of PVs [47]. Accumulating evidence indicates that the pathogenesis of FA gene-dependent diseases is closely related to excessive inflammatory activity and cellular apoptosis induced by proinflammatory cytokines. Thus, the cytokine milieu plays a pathological role in the onset and progression of FA [57,58,59]. Nevertheless, to date, understanding of the underlying molecular background has not been fully cleared. Hu et al. demonstrated that FA stem cells are highly reactive to inflammatory stimuli. The experiment based on the suppression of proinflammatory IL-1β led to a limitation of the BM failure [57]. Inflammatory-associated milieu and oxidative genotoxic stress are believed to underlie the BMF development in FA patients [58,60]. Moreover, DNA damage accumulates in FA-derived hematopoietic stem cells (HSCs), leading to BMF [61]. In addition, FA-derived cells are characterized by aldehyde accumulation and subsequent aldehyde-induced damage, leading to impaired aldehyde detoxification and excessive production of proinflammatory cytokines [1,62].

In physiological conditions, bone marrow (BM) niche provides an appropriate microenvironment to maintain HSCs at a dormancy state. At this state, HSCs remain ready for the self-renewal, proliferation and differentiation necessary to maintain hematopoietic homeostasis [63,64]. The transition of HSC from a dormant phenotype into an active form is mediated by oxidative stress and inflammatory stimuli. Thus, prolonged activation due to a hypersensitivity of the BM to inflammatory cytokines (IL-1, IL-6, tumor necrosis factor α (TNF-α), IFN-γ) leads to the HSC exhaustion (low proliferation rate and apoptosis of HSCs) and as a consequence, progressive BMF [31,65,66,67,68]. The loss of HSC quiescence, BMF and/or leukemogenesis are also linked to excessive activation of the NF-κB pathway [58]. NF-κB is a well-established factor controlling cell cycle, including proliferation, differentiation and survival [63]. Furthermore, pro-metastatic potential of cancer cells, cancer-dependent genomic instability and resistance to cytotoxic drugs are linked to constitutive NF-κB stimulation [69]. Hematopoietic homeostasis is maintained by Notch signaling. Abnormalities of the Notch pathway contribute to the development of many diseases, including hematological and solid cancers. Du et al. noted that disturbed HSC dormancy and impaired HSC self-renewal capacity were caused by TNF-α activation, which enhances Notch signaling in *Fanca^−/−^* and *Fancc^−/−^* CD150^+^CD48^−^ Lin^−^Sca1^+^c-kit^+^ (LSK) cells. Interestingly, they observed that HSC dormancy and self-renewal capacity are gradually reinstituted by downregulating NF-κB or Notch signaling in FA-derived HSCs [63]. It is worth emphasizing that there is a mutual stimulating interaction between TNF-α, NF-κB pathway [60,63] and Notch signaling [63]. Additionally, IL-1β, IL-6, IL-12p40 are released via NF-κB mediation, since it is a vital transcription factor of M1 macrophages [70]. Briot et al. noted that enhanced NF-κB transcriptional function, hyperactivity of all three mitogen-activated protein kinases (MAPKs) pathways, abundance of matrix metalloproteinase 7 (MMP-7) and TNF-α are strongly related to downregulation of *FANC* repair pathway. They found that in FA models, TNF-α may contribute in the BMF, cellular vulnerability to DNA damage, as well as the spontaneous chromosome fragility [60]. Interestingly, both the oxidative genotoxic stress and proinflammatory state led to DNA damage, which can subsequently cause excessive TNF-α secretion either directly or via stimulation of NF-κB signaling [31,63]. It is suggested that TNF-α-induced ROS (singlet oxygen (^1^O_2_), superoxide (O_2_^•−^), hydroxyl (HO^•^) and hydroperoxyl (HO_2_^•^) production plays an important role in cell cycle regulation [31]. Li et al. employed *Fancc^−/−^* murine hematopoietic stem and progenitor cells, and demonstrated that BMF develops due to TNF-α oversecretion, and leukemic transformation is caused by prolonged exposure of cells to TNF-α. The authors summarized their study that TNF-α-dependent inflammation is one of the most health-threatening stress conditions leading to FA-dependent leukemogenesis [71]. Furthermore, Du et al. claimed that abnormal Notch and NF-kB signaling pathways in FA-derived cells and modification of the BM environment are caused by an excess of TNF-α [63]. Additionally, Brégnard et al. confirmed that an imbalance in proinflammatory cytokines production leads to BMF, but cancer development in FA patients can also be mediated by prolonged IFN-α and IFN-β secretion [15]. Furthermore, the FA hematopoietic cells are characterized by the proinflammatory cytokine hypersensitivity, and this effect is intensified by anomalies in FA macrophages [72].

Additionally, a long-term inflammation leads to an insufficient self-renewal capacity of HSCs, dysregulation of the BM niche, and consequently FA-mediated BMF [31,65,66]. Thus, the limitation of pathological inflammation by deletion of transforming growth factor-β (TGF-β) or p53 prevents the BMF via the promotion of HSC cycling [66,73]. Cumulatively, we speculate that the well-known background of the hypersensitivity of FA-derived cells to proinflammatory cytokines opens the space for further study focusing on the inhibition of inflammation pathways to improve future outcomes of FA patients. Future studies should also be designed to reduce signaling pathways that increase the production of proinflammatory cytokines.

### 5.3. Destabilization of Cellular Homeostasis Due to the Oxidative Genotoxic Stress

**Figure 4:**
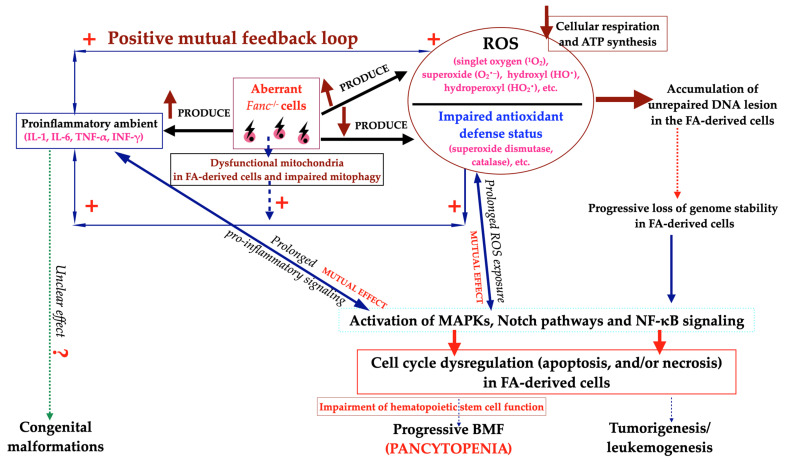
Schematic representation of how biallelic pathogenic variants in *FANC* (*Fanc^−/−^)* genes trigger a vicious circle between proinflammatory cytokines, oxidative genotoxic stress and MAPK, Notch as well as NF-κB signaling pathways.

Oxidation–reduction (redox) balance, a fundamental component of physiological processes that preserves the cellular homeostasis, is strictly based on the balance between prooxidant markers and the accompanying protective effect of antioxidant systems. The consequence of substantial alterations in the redox equilibrium, due to the associated intensification of prooxidants accumulation and simultaneous insufficient antioxidant defense mechanisms, is oxidative stress, which poses a threat to the host, through intracellular damage that is harmful to cellular structure and properties [32,74]. It is worth mentioning that various cells with the *FANCC* pathogenic variants are characterized by the production of ROS and cell apoptosis, which strongly influences the clinical phenotype of FA [75]. Additionally, in vitro and in vivo studies confirmed that ROS are generated via activation of proinflammatory cytokines excessively released by FA cells. Chronic exposure to a proinflammatory milieu also attenuates the antioxidant defense mechanisms, which generate large numbers of inflammatory-induced free radicals, leading to a significant increase in oxidative stress [31,76]. Furthermore, ROS promotes the inflammatory process by initiating various ROS-sensitive intracellular signaling pathways, including MAPK and NF-κB. Additionally, due to increased NF-κB activity, ROS supports decompensation of antioxidant defense mechanisms [77]. Reduced antioxidant potential is closely related to the clinical and cellular phenotype of FA [61]. Furthermore, Li et al. reported that the FANC pathway mediates the response to oxidative stress and cellular antioxidant defense [32]. These observations confirm the simultaneous existence of oxidative stress and inflammation in the nature of FA, that tightly influence one another (Figure 4). Interestingly, impairment of antioxidant defense mechanisms was associated with the suppression of hematopoiesis in *Fancc^−/−^Sod^−/−^* mice models [78].

Under physiological conditions, ROS are produced during mitochondrial energy metabolism. Inactivation of the FA pathway may lead to mitochondrial dysregulation, expressed by diminished ATP production, which may underlie the susceptibility to BMF and leukemogenesis in FA patients [24,79]. Furthermore, the loss of mitochondria integrity determines another source of redox stress that fuels oxidative damage and further inflammation in FA patient-derived cells, which presumably is the result of defective mitophagy [11,55]. Shyamsunder et al. confirmed that in FA-derived cells, a mitochondrial fission-dependent suppression of mitophagy occurs [55]. Sumpter et al. reported that defective mitophagy is linked to the knockdown of *FANCC, -F* or *-L,* suggesting that FANC proteins are important in eliminating dysfunctional mitochondria and excreting ROS generated by mitochondria [80]. Cappelli et al. claimed that FA cells are unable to use aerobic metabolism as their primary energy pathway, which may imply an abnormal metabolic maturation during the differentiation from HSCs to lymphocyte. The authors suggest that the increased oxidative stress in FA may be important for cells attempting to differentiate, suggesting that mitochondrial dysfunction, is a result of the genetic defect in FA [81].

Briot et al. claimed that FA cells persist constantly in harmful milieu due to the oxidative genotoxic stress and high number of DNA damage, which consequently enhance the MAPK pathways and NF-κB signaling. MAPK stimulation then triggers a vicious circle by modifying MMP-7 expression, leading to overproduction of TNF-α. This molecular vicious circle translates into the clinical picture of FA, since overstimulation of MAPK promotes the BMF, MDS and leukemia development. Briot et al. suggest that intensification of leukemic cells migration and invasion is mediated by increased MMP-7 expression [60]. Taken together, the above results indicate an impairment of systemic antioxidant defense and further support the existence of systemic oxidative stress, which influences the progression of FA. Deficiency in *FANCA*, *FANCC* and *FANCD2* genes is associated with impaired mitochondrial ROS-scavenging potential, leading to low oxygen consumption and reduced mitochondria function, due to the low ATP production [51].

### 5.4. Graphical Summary of the Vicious Cycle Between Proinflammatory Cytokines, Oxidative Stress and Upregulated Signaling Pathways in Aberrant Fanc^−/−^ Cells

Aberrant *Fanc^−/−^* cells release numerous proinflammatory cytokines and ROS leading to the accumulation of unrepaired DNA lesion in the FA-derived cells [6,12,15,24,31,58,60,61,67]. Furthermore, the overproduction of ROS with simultaneous impairment of antioxidant defense leads to deterioration of cellular respiration and reduction in ATP synthesis [1,51,52]. The excessive proinflammatory cytokine release is strongly associated with persistent genomic instability [15]. Dysfunctional mitochondria in FA-derived cells enhance the proinflammatory environment via the secretion of ROS. Furthermore, impaired mitophagy results in increased generation of ROS and proinflammatory cytokines, which contribute to the impairment of HSC function and the development of progressive BMF and genomic instability, which may further enhance the tendency for cancer development. Both proinflammatory environment and redox stress stimulate the MAPKs, Notch, NF-κB pathways and thus suppress hematopoietic homeostasis [80]. Despite numerous studies on the pathogenesis of FA, many questions are still to be answered in FA pathogenesis: Which came first, the oxidative stress or the inflammation? The first perspective covers the issue if aberrant *Fanc^−/−^* cells overproduce ROS as the initial event, soon after inflammation will develop, which will further exacerbate oxidative stress. The second facet, if inflammation develops as the first condition, then soon oxidative stress evolves, which will further intensify inflammation in *Fanc^−/−^* cells (Figure 4). Therefore, further research is needed in this area to answer this urgent question.

## 6. A Brief Summary of the Up-to-Date Facts Provided by This Manuscript

1. Presents a schematic concept of Fanconi anemia pathogenesis.
2. Presents a clear, extensive, tabular presentation of the FANC gene library with the frequency of pathogenic variants, chromosomal loci, inheritance pattern and FA protein functions.
3. Briefly describes diagnostic procedures, their applications, advantages and limitations in FA.
4. Discusses most representative procedures for treating patients with FA, their indications, advantages and limitations.
5. Provides a broad analysis of destabilization of cellular homeostasis due to disruption of autophagy process, proinflammatory state and the oxidative genotoxic stress.
6. Presents a graphical overview of the vicious circle between proinflammatory cytokines, oxidative genotoxic stress and the MAPK, Notch and NF-κB signaling pathways that operate in FA-derived cells.
7. Indicates five research directions that should be explored and developed in the context of the treatment of patients with Fanconi anemia.

## 7. Conclusions and Future Perspectives

Our literature review highlighted the current understanding of the interactions between inflammation and redox imbalance underlying the progressive BM failure in FA. Each of the characterized processes—impaired autophagy, inflammation, oxidative stress, the MAPK, Notch and NF-κB signaling pathways—is of crucial importance; furthermore, each propels the others, leading to the progressive depletion of HSCs. A solid understanding of the molecular associations between aberrant FA pathway nature and the hyperactivity of inflammation, oxidative stress in the context of FA pathogenesis, cancers and BMF will be a remarkable field for further research.

Further improvement in the understanding of the FA nature should help to design future studies, preferably in multicenter settings, that should investigate and develop:

1. A way to restore oxidative balance or increase the efficiency of superoxide dismutase and catalase.
2. A way to suppress the proinflammatory cytokines activity.
3. A way to restore the mitochondria metabolism.
4. A way to improve the functioning of the autophagy process.
5. A way to apply the natural redox regulators, including quercetin, green tea (catechins), resveratrol, etc. in adjuvant therapy in FA patients.

## Figures and Tables

**Table 1:** Current status of the *FANC* gene library with the frequency of pathogenic variants, chromosomal loci, inheritance pattern and FA protein functions.

*FANC* Gene Name (Synonymous Name)	PV Frequency	Chromosomal Locus	Inheritance Pattern	FANC Protein Function/Enzymatic Activity
*FANCA*	64%	16q24	AR	Partner of FANCG; contains nuclear localization signal
*FANCB*	2%	Xp22.31	XLR	Partner of FANCL; contains nuclear localization signal
*FANCC*	12%	9q22.3	AR	Partner of FANCE
*FANCD1 (BRCA2)*	2%	13q12.3	AR	Involved in HR; facilitates Rad51 function together with BRCA1 and PALB2
*FANCD2*	4%	3p25.3	AR	Form the ID2 subcomplex with FANCI
*FANCE*	1%	6p21.3	AR	Partner of FANCC and FANCD2; contains nuclear localization signal
*FANCF*	2%	11p15	AR	Forms the subcomplex with FANCC and FANCE
*FANCG (XRCC9)*	8%	9p13	AR	Partner of FANCA
*FANCI (KIAA1794)*	1%	15q25-26	AR	Partner of FANCD2; monoubiquitinated and phosphorylated following DNA damage
*FANCJ (BRIP1)*	2%	17q22.3	AR	5′-to-3′ helicase
*FANCL (PHF9)*	0.4%	2p16.1	AR	Partner of FANCB
*FANCM (Hef)*	0.1%	14q21.3	AR	Translocase
*FANCN (PALB2)*	0.7%	16p12.1	AR	Involved in HR; facilitates Rad51 function together with BRCA1 and BRCA2
*FANCO (RAD51C)*	0.1%	17q25.1	AR	Strand-transfer protein; involved in recombinational repair of damaged DNA and in meiotic recombination
*FANCP(SLX4)*	0.5%	16p13.3	AR	Structure specific endonuclease subunit
*FANCQ (ERCC4)*	0.1%	16p13.12	AR	NER nuclease; forms the complex to cleave ICLs
*FANCR (RAD51)*	~<0.1%	15q15.1	AD	Recombinase required for strand invasion
*FANCS (BRCA1)*	0.1%	17q21.31	AR	Involved in HR; facilitates Rad51 function together with BRCA2 and PALB2
*FANCT (UBE2T)*	0.1%	1q32.1	AR	E2 ubiquitin-conjugating enzyme for the E3 ubiquitin ligase FANCL
*FANCU (XRXX2)*	0.1%	7q36	AR	RAD51-like recombinase
*FANCV (MAD2L2/REV7)*	One patient	1p36	AR	Polymerase Pol ζ subunit
*FANCW (RFWD3)*	One patient	16q23.1	AR	E3-ubiquitin ligase

AD: autosomal dominant; AR: autosomal recessive; XLR: X-linked recessive, HR: homologous recombination, NER: nucleotide excision repair.

**Table 2:** Brief summary of FA diagnostic procedures, their brief description, advantages and limitations.

Methods	Brief Description	Advantages	Limitations
Chromosome breakage test in peripheral blood lymphocytes and/or fibroblasts	Elevated baseline of MMC/DEB-induced chromosome breakage compared to the control sample followed by average number of aberrations per cell and per aberrant cell and percentage of cells with radial figures.	1. The presence of radial figures is considered the hallmark of the FA. 2. Analysis of baseline breakage allows the diagnosis of other chromosome instability syndromes with specific chromosomes abnormalities. 3. Positive result of test in fibroblasts confirms the diagnosis in patients with strong evidence of FA but negative or equivocal peripheral blood chromosome breakage test.	1. Difficulties in stimulation is proliferation of cultured cells and in harvesting a sufficient number of metaphase spreads to analyze in patients with a low number of lymphocytes. 2. The risk of cell culture failure. 3. The technique is time-consuming, costly and requires a well-qualified staff. 4. Test can produce false-negative and false-positive results because of no 100% specificity. 5. Follow-up molecular testing should be performed to identify the patient’s pathogenic variant in *FANC* genes.
Flow cytometry (Cell cycle assay)	In the analysis of the cell cycle of lymphocytes/fibroblasts in FA patients, an increased number of cells arrested in the G2/M phase after culture with a DNA cross-linking compound, e.g., MMC or DEB, is observed.	1. Rapid screening method. 2. It is used for the differential diagnosis of patients with FA and other genetic instability syndromes. 3. Fibroblasts analysis is applicable in the diagnostics of hematopoietic somatic mosacism.	1. The assay represents low specificity. 2. Negative results may appear in FA patients who developed MDS or AML.
Multiplex ligation-dependent probe amplification (MLPA) or array Comparative Genome Hybridization (aCGH)	Detection of copy number variants (CNVs)	1. Highly sensitive and high throughput methods. 2. Detection of all large deletions, duplications and insertions.	1. It is not applicable in the diagnosis of somatic mosaicism. 2. Broad, large MLPA panels are often not comprehensive for each of the syndromes they analyze, so an FA-specific panel is still preferred.
Dedicated NGS gene panel	Targeted panels identify novel PVs within known *FANC* genes	1. The test is performed to confirm the diagnosis. 2. Clinically important genes associated with a patient phenotype are analyzed in a single test. 3. Fast turnaround time and lowest cost option. 4. CNV analysis can be included in an assay with point mutation NGS identification.	1. It is not applicable in the diagnosis of somatic mosaicism. 2. Does not detect larger deletions or duplications if copy number analysis is not included. 3. May fail to detect deep intronic variants or variants in gene promoter regions. 4. Variants of unknown significance (VUS) may be identified. 5. Incidental discovery of a hereditary cancer risk not associated with the underlying FA diagnosis is possible.
Whole genome sequencing (WGS)/Whole exome sequencing (WES)	Can identify novel *FANC* genes in contrast to dedicated panel tests which screen specific regions of the genome.	1. All coding and non-coding regions (exons, introns and regulatory regions) of the genome are sequenced in a single test. 2. May provide value for patients with no PVs identified on dedicated panel testing or WES (may provide opportunity for gene discovery through research). 3. Can provide information for conditions other than FA if the diagnosis is uncertain.	1. Standards of what defines a clinical genome are still emerging. 2. Assay cost, turnaround time and variant interpretation require further refinement for WES to be clinically relevant. 3. May uncover findings unrelated to the patient’s diagnosis, with the potential for a greater number of VUS than panel testing and WES.

**Table 3:** Most representative procedures for treating patients with FA, their indications, advantages and limitations.

Methods	Indications	Advantages	Limitations
Hematopoietic stem cell transplantation (HSCT)	1. Severe pancytopenia (BMF due to severe aplastic anemia) or progression of moderate cytopenia, poor prognostic cytogenetic aberrations and overt MDS/AML.	1. The only curative procedure for the hematological complications, including AML. 2. Improves life expectancy and quality of life.	1. High risk of developing solid tumors. 2. Elderly, comorbidities, no donors. 3. High risk of graft-versus-host disease (GVHD) and immune dysfunction. 4. Finding unrelated donor. 5. Restrictive requirements for pre-transplant period.
Androgene Therapy	1. Patients who are not eligible for HSCT at a given time.	1. Highly effective in stabilizing blood counts. 2. Broad spectrum of action, including pleiotropic effects on erythropoiesis, telomere regulation, immune homeostasis, maintenance of the musculoskeletal, cardiovascular, reproductive and neural.	1. High risk of developing MDS and AML. 2. Short-term response to treatment or temporary response in some patients. 3. An intervention of questionable effectiveness, dependent on the dose used. 4. Probably more effective in improving hemoglobin levels and variable neutrophil and platelet response. 5. Therapy elevates liver function tests. 6. Side effects, including virilization.
Gene Therapy	1. So far, only applicable for genotype FANCA. 2. To improve BM function.	1. Highly effective if applied at the early stage of BMF. 2. Alternative treatment to (HSCT).	1. Limited use due to vector capacity. 2. Collection of an adequate number of HSPCs from patients with FA. 3. Risk of cellular toxicities and insertional mutagenesis with lentiviral complementation strategy. 4. High costs linked to designing of GMP-compatible viral vectors. 5. Clinical trials with limited study population. 6. Ongoing clinical trials with still a short follow-up.
Novel targeted chemoprevention therapy	1. Natural flavonoids (quercetin) with antioxidant properties and positive effects on hematopoiesis. 2. Treatment based on ROS reduction is indicated to diminish the risk of cancer in FA. 3. Resveratrol, another antioxidant agent, has been shown to improve hematopoiesis in Fancd2^−/−^ mice.	1. Safe and well-tolerated treatment. 2. Reduction of peripheral blood ROS, and consequent lipid peroxidation. 3. Strong evidence that it reduces the TNF-α-generated by ROS. 4. High probability to prevent or ameliorate BM failure. 5. Animal models have shown that FA-derived cells do not transform into leukemia after treatment. 6. Quercetin increases HSCs reserves. 7. Good toxicity profile 8. Protects against the toxic effects of DNA crosslinkers. 9. Suppresses NF-κB signaling and aldehyde-mediated oncogenesis.	1. The lack of side effects. 2. Clinical trials with limited study population. 3. Ongoing clinical trials with still a short follow-up.
